# Case report: Large-size intramuscular nodular fasciitis, a challenging histopathologic diagnosis confirmed by molecular detection of USP6 gene rearrangement: Case report and literature review

**DOI:** 10.3389/pore.2023.1610785

**Published:** 2023-01-20

**Authors:** Changrong Wang, Wei Wang, Rujun Xu, Jingjing Xiang

**Affiliations:** Department of Surgical Pathology, Affiliated Hangzhou First People’s Hospital, Zhejiang University School of Medicine, Hangzhou, Zhejiang, China

**Keywords:** nodular fasciitis, gene rearrangement, large-size, intramuscular, USP6

## Abstract

The intramuscular subtype of nodular fasciitis (NF) is rare with lesions normally not more than 2 cm in size and characterized by pseudosarcomatous morphology. We report a case of a 27-year-old man with a large-size intramuscular NF. The patient came for treatment complaining of an increasingly enlarged mass in the left upper arm for 4 months. Magnetic resonance imaging (MRI) confirmed the presence of a well-defined tumor measuring 5 cm within the outer edge of the middle humerus. Microscopically, the neoplasm was rich in fibroblasts and myofibroblasts in an interlaced pattern with high mitotic index and evident multinuclear giant cells. Erythrocyte extravasation was easily seen in the stroma. The tumor border was infiltrative. Immunohistochemically, the tumor cells were positive for smooth muscle actin (SMA) and negative for cytokeratin, desmin, H-Caldesmon, CD34, S100, ALK, and *β*-catenin. Fibrosarcoma was highly suspected by histopathological and immunohistochemical examination. Molecular detection demonstrated evidence of ubiquitin-specific peptidase 6 (USP6) gene rearrangement in this tumor. Based on the findings, the tumor was diagnosed as intramuscular NF. At 56 months after the initial surgery, the patient had recovered with no evidence of recurrence or metastasis. Large-size intramuscular NF is very rare and easily overdiagnosed as malignant tumor due to its obvious pseudosarcomatoid pathological features. USP6 gene rearrangement detection can effectively avoid this major misdiagnosis.

## Introduction

Nodular fasciitis (NF) is a self-limiting fibrous neoplasm harboring the fusion gene myosin heavy chain 9/ubiquitin-specific peptidase 6 (MYH9-USP6) as a recurrent somatic gene fusion event, as described in the current World Health Organization classification [1-5]. NF is mainly divided into three subtypes based on anatomical location: subcutaneous (most common), fascia, and intramuscular [6]. Of these, intramuscular NF is a rare subtype sharing similar clinical, histologic, and immunohistochemical features, as well as the gene expression profile, with subcutaneous NF[6]. Normally, the lesions of intramuscular NF are less than 2 cm in size, but occasionally can be > 4 cm, which can be easily over-diagnosed as a soft tissue sarcoma because of the “sarcoma-like” morphology. Here, we present a case of intramuscular NF on the upper arm of a young man with a lesion 5 cm in size that was initially suspected as a sarcoma and diagnosised by detecting the ectopic rearrangement of USP6 gene.

## Case presentation

**FIGURE 1:**
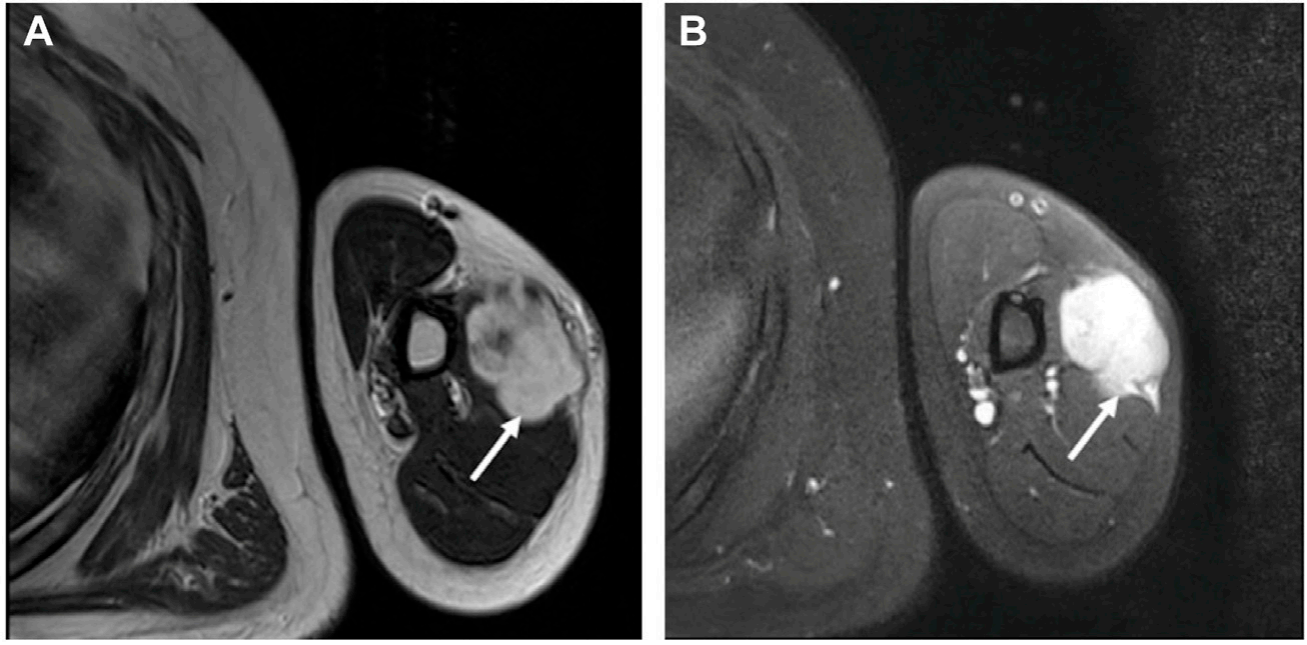
MRI revealed an irregularly well-defined nodule at the outer edge of the middle humerus. **(A)** The nodule (the arrow) had slightly enhanced signal intensity on T1-weighted imaging. **(B)** On T2-weighted imaging, there was significantly enhanced heterogeneous high signal intensity (the arrow).

A 27-year-old man came for treatment complaining of a progressively enlarging mass on his left upper arm over 4 months with occasional slight discomfort. He reported no history of trauma, infection, or prior surgery of the lesion. Magnetic resonance imaging (MRI) confirmed the presence of a well-defined mass located deeply in the outer edge of the middle humerus muscle, but at the focal border, it had protruded into the surrounding muscle. The signal intensity was slightly enhanced on T1-weighted imaging (Figure 1A) and marked heterogeneous enhancement on T2-weighted imaging (Figure 1B). The MRI findings suggested the possibility of fibromatosis. The patient underwent surgery for mass excision. On gross examination, it was an irregular, solitary mass measuring 5.0 cm × 4.0 cm × 4.0 cm, in the muscle with a grayish-white cut surface.

**FIGURE 2:**
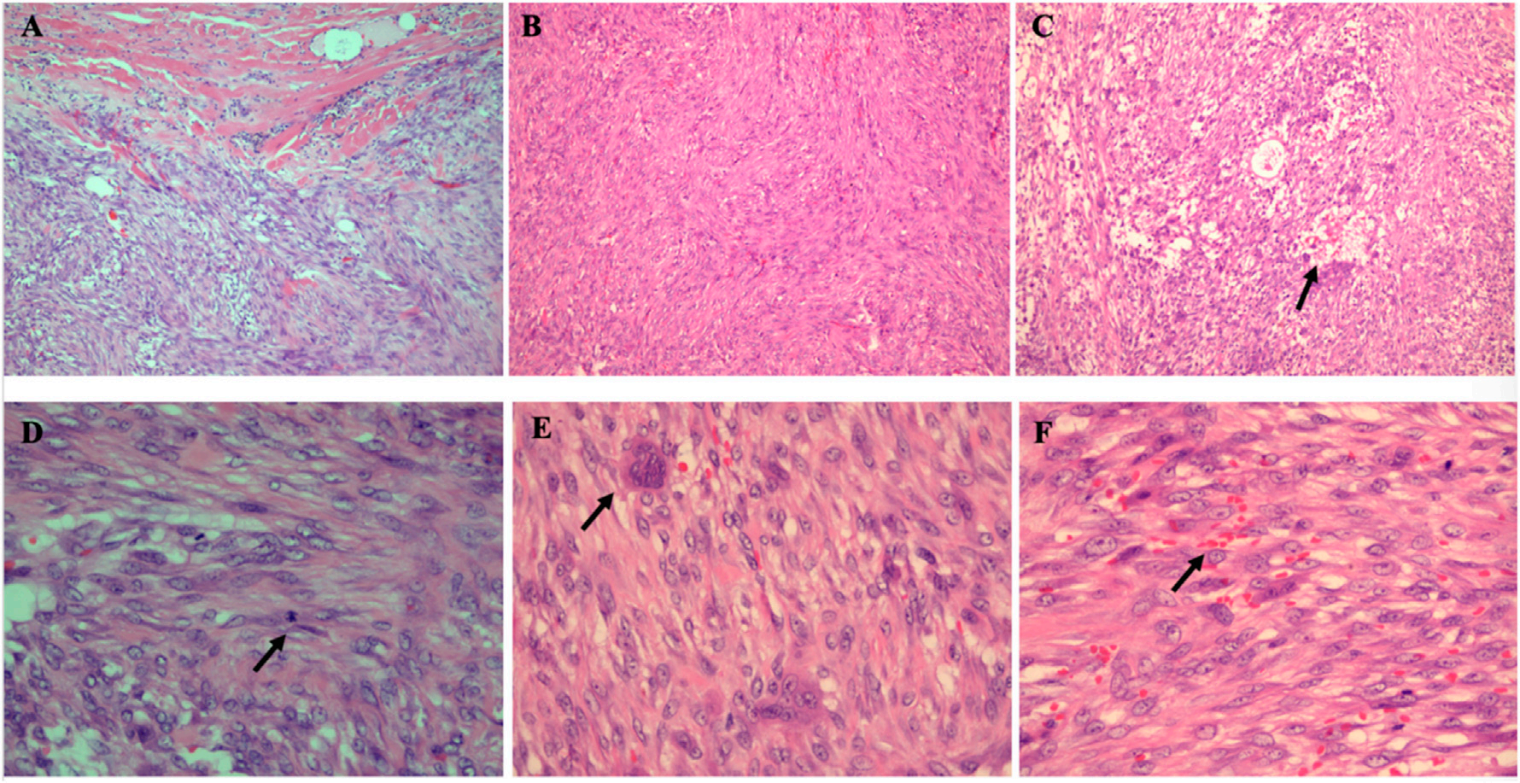
Histopathological findings of the intramuscular tumor. **(A)** Tumor cells infiltrating into the surrounding skeletal muscle (HE× 100). **(B)** Tumor cells arranged in an interlaced pattern (HE×40). **(C)** In the scarce cell area, there were microcystic changes (the arrow) (HE× 100). **(D)** Mitotic figures were high (the arrow) (HE× 400) **(E)** Multinucleated cells were conspicuous (the arrow) (HE× 400). **(F)** The tumor showed erythrocyte extravasation (the arrow) in the stroma (HE× 400).

Microscopic examination showed that the tumor was relatively well demarcated, but in the focal, it was infiltrating the adherent muscle (Figure 2A). It was highly cellular and composed of fibroblasts and myofibroblasts arranged in bundles with an interlaced pattern (Figure 2B). Part of the lesion was discohesive and myxoid with a microcystic appearance (Figure 2C). Some tumor cells had mild atypia with active mitotic counts (the average mitotic counts up to 8 per 10 high-power fields, Figure 2D) and mingled conspicuous multinuclear or atypical giant cells (Figure 2E). Erythrocyte extravasation was easily seen on a background of collagenous stroma (Figure 2F), with some hemosiderin deposition and scattered lymphoplasmacytic cells infiltration.

**FIGURE 3:**
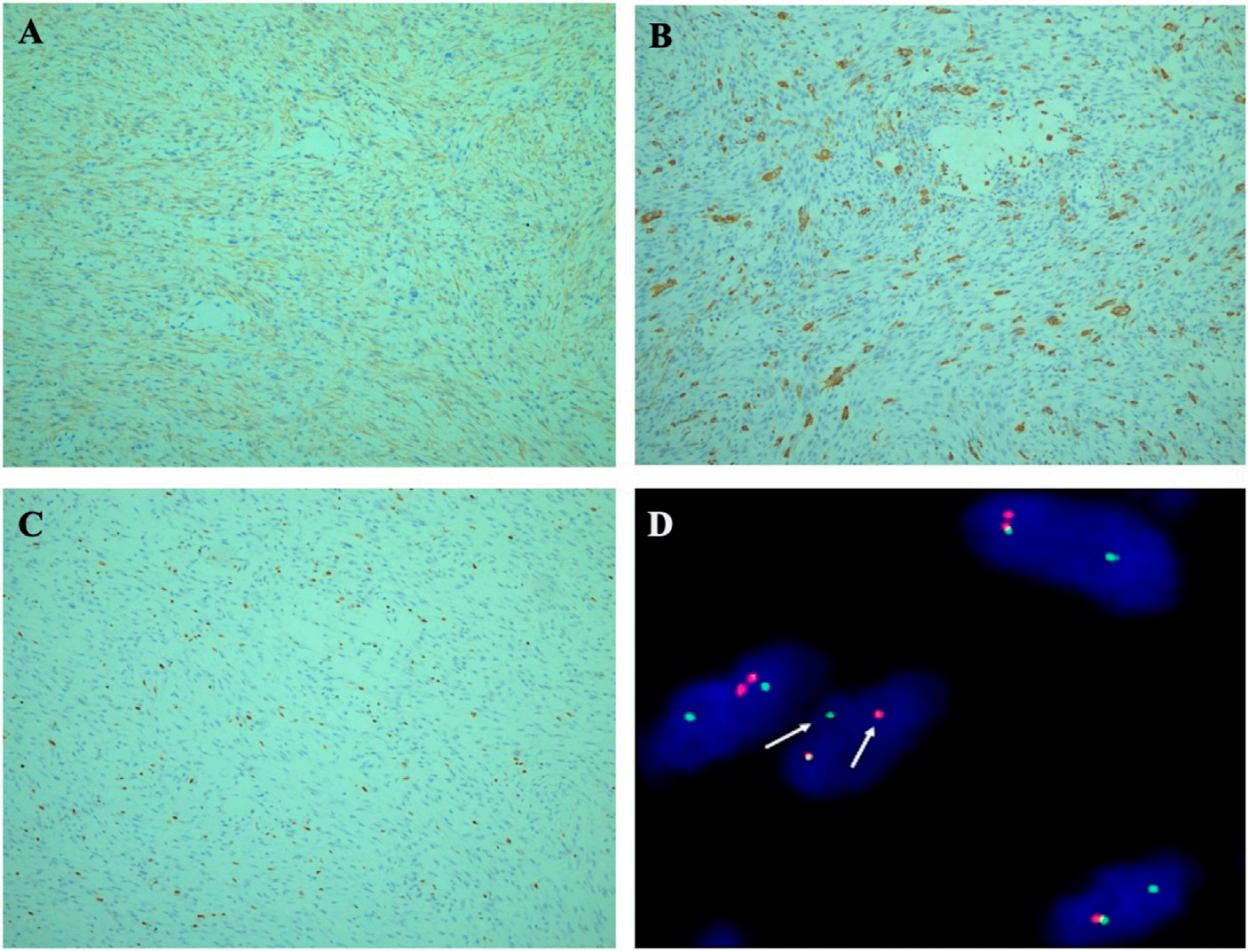
**(A)** Immunohistochemical staining. Tumor cells were strongly immunoreactive for SMA. (×100). **(B)** The multinuclear giant cells were positive for CD68 (× 100). **(C)** Ki-67 proliferation index was 10% (×100). **(D)** FISH illustrated rearrangement of the USP6 gene locus 17p13 using a break-apart probe set, with separation of red and green signals (the arrow).

Imunohistochemical detection, The tumor cells were only positive for smooth muscle actin (SMA) (Figure 3A), negative for cytokeratin, desmin, H-Caldesmon (see supplement data), CD34, S100, ALK, and *β*-catenin. The multinuclear giant cells were positive for CD68 (Figure 3B)). The average rate of ki67 positivity was 10% (Figure 3C). According to histopathological and immunohistochemical studies, fibromatosis was first excluded for the tumor’s growth pattern and *β*-catenin negative. Leiomyosarcoma was ruled out as well for H-Caldesmon and desmin negative. However, it was still difficult to distinguish the tumor from a (myo) fibroblastic sarcoma.

Molecular detection was performed on 4 μm paraffin-embedded tumor section by Fluorescence *in Situ* Hybridization (FISH) using a USP6 dual-color break-apart probe (Guangzhou LBP Medicine Science and Technology Co., Ltd., Guangzhou, China). The FISH results revealed evidence of USP6 gene rearrangement given the division of green and red signals (Figure 3D). The average percentage of USP6 split signals was 40%. Molecular findings confirmed the diagnosis of intramuscular NF. At 56 months after surgery, the patient recovered well with no evidence of recurrence or metastasis by the imaging examination.

## Discussion

Intramuscular NF is a rare benign fibrous tumor, accounting for only 5.9% of 272 NF cases in a previous retrospective study [6]. Unfortunately, there is limited datas about intramuscular NF, particularly regarding the clinicopathological features. As far as we know, until now, only seven cases of intramuscular NF have been reported, including our case, in the English literature *via* a search of the PubMed database [7-12]. All seven cases are reviewed and summarized in Table 1. Although there was no predominant gender difference (male to female ratio, 3:4) among these seven cases, there was a wide age range from 11 to 46 years (mean age, 32.5 years). Tumors were mainly found in deep locations of the limbs and trunk, including the thigh, rectus abdominis muscle, gluteal region, right axillary tail, erector spinae muscle, neck, and upper arm. The preoperative duration was relatively short, no more than 2 months in four cases, while our case was 4 months, and the other two were over 1 year in duration. All seven patients complained of pain, from mild to severe. Other clinical symptoms included nerve compression, swelling, and numbness. The patient in this report just felt a little discomfort, but the mass in the upper arm gradually increased. The follow-up periods ranged from 6 months to 10 years. Five patients recovered with no evidence of recurrence or metastasis. However, one patient developed multiple recurrences and ultimately metastasis at 10-year follow-up, which was caused by inadequate surgery for the complexity of the deep anatomical location and poorly infiltrated border. Fortuntely, our patient had been followed up 56 months with no signs of recurrence by the adequate surgery. Grossly, the maximum dimensions ranged from 1.8 to 10 cm (median, 4.9 cm) with grayish-white and solid cut surfaces. The lesions in three cases were >4 cm in size with less defined borders including our case.

**TABLE 1:** Clinicopathological and genetic features of the current and previously reported cases of intramuscular NF.

Case [Ref]	Age (years)/Gender	Preoperative symptom	Preoperative duration (months)	Tumor location	Tumor size (cm)	Tumor border	Molecular genetics	Follow-up (months)
1 [7]	11/F	Occasional discomfort	2	Rectus abdominis muscle	2.6	Well-circumscribed	ND	NEOD after 6
2 [9]	19/M	Dull ache and swelling	24	Left gluteal region	10	Locally infiltrative margin	ND	NEOD after 6
3 [10]	31/F	Persistent pain	2	Right axillary tail	1.8	Well-circumscribed	ND	NR
4 [8]	42/F	Nerve compression, severe pain and numbness	12	Proximal right thigh	2.5 to 9.7	Extensively infiltrative margin	PPPR6-USP6 gene fusion	Four times recurrences and eventually local multiple metastasis during 120
5 [11]	46/M	Back pain	1	Right erector spinae muscle	2.8	Well-circumscribed	ND	Spontaneous regression after 1
6 [12]	45/F	Neck swelling	2	over right side of neck	3	Partially capsulated and surrounded by skeletal muscle fibers	ND	NEOD after 6
7 present case	27/M	Little discomfort	4	Left upper arm	5	Locally infiltrative margin	USP6 gene rearrangement	NEOD after 56

Microscopically, most cases had hyper- and hypocellular areas with a focally myxoid or microcystic appearance. The predominant growth pattern in the hypocellular areas were S- or C-shaped fascicles, or storiform-like. The tumor cells all were fibroblasts or myofibroblasts with plump and regular spindle-shaped nuclei lacking hyperchromatism and pleomorphism. Mitotic figures were active, but no atypical forms were present. Most cases had a typical background of collagen and erythrocyte extravasation with or without lymphocyte infiltration and hemosiderin deposition. However, in distinct contrast, in the present case, (myo) fibroblasts were mildly atypic with many multinuclear giant cells mixed, it was extremely mimic soft tissue sarcoma. Immunohistochemically, the immunophenotype of the tumor cells was (myo) fibroblastic expressing SMA.

USP6 (17p13), a deubiquitinating protease involved in cell trafficking, protein degradation, signaling, and inflammation, is a valuable adjunct for the diagnosis of intramuscular NF. Initially, a chromosome 17p13 rearrangement was observed as an oncogenic-activated event in aneurysmal bone cysts [13-18]. In 2011, Erickson-Johnson et al. observed that the MYH9 promoter region fused with the entire coding region of USP6, which resulted in overexpression of USP6 in NF [1]. Based on this observation, they were the first to put forward a new term of transient neoplasia induced by MYH9-USP6 gene fusion in NF. In 2014, a study by Oliveira et al. confirmed that detection of USP6 genomic rearrangements was valuable for the diagnosis of NF, since they had observed high sensitivity and specificity (respectively 93% and 100%) using FISH for USP6 in their clinical practice [3]. Subsequently, several other studies also found that USP6 rearrangements in the NF has the same characteristics [4, 5]. However, in 2017, a study by Patel et al identified seven other novel pathogenic fusions of USP6 in NF by RT-PCR, including RRBP1, CALU, CTNNB1, MIR22HG, SPARC, THBS2, and COL6A2 [19]. Thus, in our clinical practice, we prefer to use the USP6 break-apart probe to detect USP6 gene translocation in NF. However, USP6 gene rearrangement is not a unique genetic alteration for aneurysmal bone cysts and NF, as it can exist in multiple lesions, including myositis ossificans, giant cell lesions of the small bones, fibromas of the tendon sheath, and fibro-osseous pseudotumors of the digits [13, 16, 20-25]. These lesions may belong to the same disease spectrum, but the clinical features, histopathological characteristics, and gene fusions differ.

A recent retrospective study reported that the percentage of USP6 break-apart FISH signals was significantly negatively correlated with preoperative tumor duration and significantly positively correlated with tumor mitotic counts [26]. Tumors with preoperative duration more than 3 months, the USP6 FISH separation signal was less than 30%, and mitotic count was low. However, in our case, preoperative duration was 4 months and the tumor USP6 split signals still reached 40%, with an active mitotic count. This may be related to the deep location and large size of the tumor.

Owing to its pseudosarcoma-like morphology, deep location, and invasive bordoer, large size intramuscular NF is easily misdiagnosed as other soft tissue sarcomas or neoplasms. A study by Erber et al in 2018 found that only 33% of 71 lesions were correctly diagnosed as NF, including intramuscular NF, and 33% were initially over-diagnosed as malignant tumors or low malignant potential tumors [5]. The main differential diagnoses include low-grade myofibroblastic sarcoma (LGMFS), which is also composed of spindle cells arranged in bundles and storiform pattern. But by contrast, LGMFS have moderate cellular atypia and diffusely infiltrative margin, local recurrence is frequent, even at multiple sites, and treatment should combine complete surgical excision with radiotherapy [27]. Intramuscular NF thus must be differentiated from LGMFS. However, both lesions show that the (myo) fibroblastic immunophenotyped, FISH detection of USP6 gene rearrangement is essential for differential diagnosis of LGMFS and intramuscular NF. Low-grade fibromyxoid sarcoma (LGFMS) is also similar to intramuscular NF, both with a background of myxoid and collagenous stroma. However, LGFMSs commonly show a mixed composition of alternately distributed collagen like and mucoid areas, and nearly 40% of LGFMSs are scattered with giant collagen rosettes [28]. LGFMS is specifically expressed MUC4 but not SMA, and FUS fusion gene exists in most cases [29]. Fibromatosis is another (myo) fibroblast neoplasm which is characterized by the proliferation of elongated, uniformly shaped spindle cells in the collagenous stroma, with obvious infiltrating borders, and shows nuclear *β*-catenin positive for the *β*-catenin (CTNNB1) gene mutation [30]. Cellular NF can be misdiagnosed as leiomyosarcoma due to more mitoses, dense fasciculation, and diffuse expression of SMA by spindle cells. However, NF did not show smooth muscle cell differentiation characteristics and was negative for desmin and H-caldesmon.

Most patients with intramuscular NF recovered well after adequate surgery, and local recurrence was quite rare following incomplete resection [8,31]. However, among the seven reported cases of intramuscular NF, one presented as a low-grade malignant tumor that developed multiple recurrences and eventually metastasized during the 10-year follow-up period. Molecular analysis showed that there was an amplified new fusion gene PPPR6-USP6 [8]. Different USP6 fusion genes may have various biological behaviors. Further research is required to confirm the relationship between the behaviors of intramuscular NFs and different pathogenic fusion partners of USP6.

## Conclusion

In summary, the clinicopathological characteristics of intramuscular NF were based on previous reports and the present case. A histopathological diagnosis of a large-size intramuscular NF is challenging due to the pseudosarcomatous morphology, larger size, deep location, and infiltrative margin. Fortunately, USP6 gene rearrangement is a valuable adjunct for the differential diagnosis of intramuscular NF and other tumors. Further investigations are required to detect different pathogenic fusion partners of USP6, which would be meaningful to expand the biological potentials of intramuscular NF.

## Data Availability

The original contributions presented in the study are included in the article/Supplementary Material, further inquiries can be directed to the corresponding author.

## Abbreviations

NF, nodular fasciitis; MYH9-USP6, myosin heavy chain 9/ubiquitin-specific peptidase 6; MRI, Magnetic resonance imaging; SMA, smooth muscle actin; FISH, Fluorescence *in Situ* Hybridization; LGMFS, low-grade myofibroblastic sarcoma; LGFMS, Low-grade fibromyxoid sarcoma.
